# Psychosocial and biological pathways to aging: The role(s) of the behavioral and social sciences in geroscience

**DOI:** 10.1007/s00391-024-02324-1

**Published:** 2024-07-10

**Authors:** Paul Gellert, Enrique Alonso-Perez

**Affiliations:** 1 Institut für Medizinische Soziologie und Rehabilitationswissenschaft, Charité – Universitätsmedizin Berlin, Charitéplatz 1, 10117 Berlin, Germany; 2 Einstein Center Population Diversity, Berlin, Germany

**Keywords:** Geroscience, Interdisciplinary, Social hallmarks of aging, Environment, Biological hallmarks, Geroscience, Interdisziplinarität, Soziale Merkmale des Alterns, Umwelt, Biologische Merkmale

## Abstract

While the biological hallmarks of aging are widely recognized as fundamental mechanisms of biological aging, more recently, there have been calls within geroscience to understand the aging process more comprehensively by adding a perspective of the social hallmarks of aging. Social and behavioral factors, such as socioeconomic status, life events or behavior and beliefs can alter the aging process per se and act in complex interactions with biological pathways. In addition, underlying biological pathways have been proposed for various psychosocial concepts, such as views on age and relationship quality. The aim of the present article is to provide narrative insights into theoretical and empirical developments between social and behavioral gerontology and geroscience or biogerontology. This article focuses on the potential of an interdisciplinary aging research but it also sets out the critical perspective that social gerontology provides.

## Abstract (de)

Während die „biologischen Merkmale des Alterns“ weithin als grundlegende Mechanismen des biologischen Alterns anerkannt sind, gibt es in jüngster Zeit in der Geroscience Forderungen, den Alterungsprozess umfassender zu beschreiben, indem man eine Perspektive der „sozialen Merkmale des Alterns“ hinzufügt. Soziale und verhaltensbezogene Faktoren wie der sozioökonomische Status, Lebensereignisse oder Verhaltensweisen und Überzeugungen können den Alterungsprozess per se verändern und in komplexen Wechselwirkungen mit biologischen Signalwegen wirken. Darüber hinaus wurden zugrunde liegende biologische Pfade für verschiedene psychosoziale Konzepte vorgeschlagen, beispielsweise für Altersbilder und die Beziehungsqualität. Ziel des vorliegenden Artikels ist es, narrative Einblicke in theoretische und empirische Entwicklungen zwischen sozial- und verhaltenswissenschaftlicher Gerontologie und Geroscience bzw. Biogerontologie zu geben. Während sich dieser Beitrag auf das Potenzial einer interdisziplinären Alternsforschung konzentriert, stellt er auch die kritische Perspektive dar, die die soziale Gerontologie bietet.

Biological aging is described as a gradual or progressive deterioration of health, including the functional impairment of physiological systems and disease, leading ultimately to death [17]. Biological hallmarks of aging ([17, 18]; see also Simm and Fuellen in this special issue) are, in particular, genomic instability, telomere attrition, epigenetic alterations, loss of proteostasis, disabled macroautophagy, deregulated nutrient sensing, mitochondrial dysfunction, cellular senescence, stem cell exhaustion, altered intercellular communication, chronic inflammation and dysbiosis. Importantly, these mechanisms are interdependent and can be altered by therapeutic interventions. Understanding the biological underpinnings of the aging process itself and being able to manipulate these, is likewise the foundation of geroscience (e.g., [12]).

## Behavioral and social science as a “vehicle” for moving from the laboratory to people

Behavioral and social science is an umbrella term for a variety of disciplines, approaches and methods. Behavioral sciences examine environmental aspects and behavioral, mental, social and physiological processes as well as the interactions of these processes with the social and natural environment, which can be understood as antecedents and consequences of behavior. Disciplines include psychology, anthropology, sociology, economics and cognitive science. Social science focuses more on the study and theorizing of societies and the social exchange processes of individuals within societies. Disciplines include sociology, anthropology, psychology, economics and political science. Behavioral and social gerontology studies how women and men adapt to the environment or shape the environment as they age through the application of behavioral and social theories to aging.

While the geroscience community has long been focused on biological mechanisms in laboratory studies using animal models, the geroscience literature is increasingly opening up to behavioral and social sciences. Behavioral and social science are then described as a “vehicle” for translation from the laboratory to the real-world settings [19]. This approach rarely considers behavioral and social science as equal contributors to the development of interdisciplinary theories of aging. Moffitt argued that geroscience can be “augmented” by behavioral and social sciences to foster the translation from animal models to humans in the living environments. Additionally, the author posited that health inequalities that could otherwise arise from geroscience research can be reduced when knowledge from behavioral and social sciences is considered. Another line of argumentation describes behavioral and social sciences as crucial for information on the design of clinical trials of geroscience interventions and outcomes [19, 21, 23].

## Linking social and behavioral gerontology with biological aging research

Social aging, as opposed to biological aging, has been conceptualized as the aging processes and outcomes that are shaped in their meaning and experiences by societal factors and by the social construction of aging [14]. Behavioral aging refers to aging-related behaviors and their antecedents and outcomes as well as the behavioral interactions between humans and their physical and social environments (for further discussion, see [26]).

A seminal work in social and behavioral aging research recently introduced the social hallmarks of aging [6]. According to Crimmins these social hallmarks are lifetime socioeconomic status, adversity in childhood and adulthood, being a member of a minority group, adverse health behavior and adverse psychological states. Crimmins’ work can be understood as a current push to define the psychosocial conditions of aging and thereby further solidify the relationships to the biological foundations of aging. While it needs to be highlighted that social determinants of individual diseases or healthy aging have been established for decades, the attempt by Crimmins tries to conceptualize the relation of psychosocial factors on the aging process per se. Furthermore, in the theoretical and empirical work of Crimmins, the interrelation of the social hallmarks of aging (i.e., age, gender, socioeconomic status, childhood trauma and health, chronic stressors, depressiveness, negative outlook, and health behavior) with biological mechanisms (i.e., bioage, epigenetic age acceleration, telomere length, mitochondrial DNA copy number) on common outcomes (i.e., instrumental activities of daily living, multimorbidity, cognitive dysfunction, and mortality) was stressed [6]. Recent advances in the availability of high-quality longitudinal panel data containing biological as well as psychosocial variables make the investigation of truly biopsychosocial research on the aging process possible (e.g., Health and Retirement Study [6]).

A recent example where the social and biological hallmarks of aging were combined into a coherent framework is the determinants of multimorbidity in the primer on multimorbidity by Skou et al. [24]. Within this framework, distinct synergistic interplays between both biological and psychosocial factors on multimorbidity were assumed. A third direct interaction with multimorbidity is assumed via the factor “medications”.

The theoretical establishment of an interplay between biological and psychosocial factors is not new. Baltes earlier proposed a meta-theory of aging which outlined the complex interplay of biological and psychosocial factors [1]. The principles of this theory entail a negative age correlation, where genome-based plasticity and biological potential decrease with age. While the biological potential decreases with age, the need for culture-based compensation of biological losses increases. Finally, the efficiency of culture decreases as humans move to very old age.

Another framework was outlined by Kuh et al. [3]. Their integrated life course model of aging combines physiological capacity (e.g., central nervous system integrity, endocrine and immunological system homeostasis, age-related changes in body composition and energy capacity and consumption) emotional health, reproductive health and physical (e.g., material conditions, pollutants, assisted technology) and social environmental factors (e.g., family, neighborhood, retirement, healthcare) and their interaction through specific phases of the life course (i.e., conception, prenatal, prepubertal, pubertal, maturity, senescence).

A fourth well-established framework on the interrelation of biological and psychosocial mechanisms is the biopsychosocial model of the International Classification of Functioning (ICF) of health and illness, which has been used in rehabilitation as well as in geriatrics and related fields. The social and biological models of the ICF are already incorporated within the framework of the comprehensive geriatric assessment for clinical practice.

**Fig. 1:**
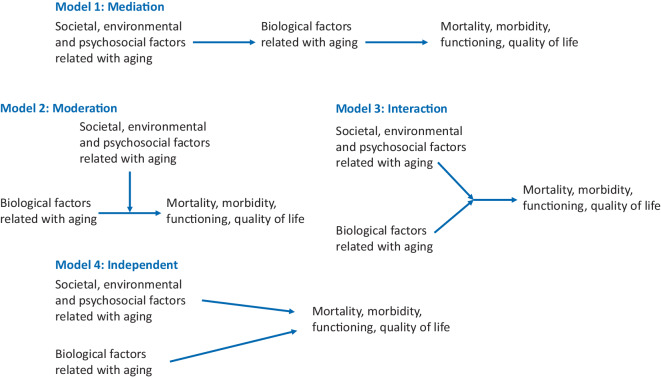
Theoretical interrelations of psychosocial and biological pathways to aging

Figure 1 outlines the theoretical interrelations between biological and psychosocial factors. A first class of models (1 “mediation”) represents a class of models that assumes an indirect effect of psychosocial factors via biological factors on health outcomes. Some models from research on age stereotypes [16, 28] and health and social exchange processes and health [4, 13] belong to this class. Another class of models (2 “moderation”) assumes a connection between biological factors and health, which can, however, be relativized by psychosocial factors. The theory of Baltes belongs to this class [1] as well as the life course model by Ben-Shlomo et al. [3]. A third class of models (3 “interaction”) assumes a dynamic interaction between both biological and psychosocial factors. This class is different from the previous class “moderation” in that an equal interplay of factors is interpreted, while in moderation the path between biology and health or illness is altered by psychosocial factors. The model according to Skou et al. [24] and the ICF model belong to this class. Finally, a fourth class of models (4 “independent”) can be assumed, which assumes the independent or direct effects of both factors, biological and psychosocial. Crimmins’ analyses [6] belong to this class, which can also be understood as a precursor to model class 1.

## Examples of established biopsychosocial aging research

While fully interdisciplinary aging research is still scarce, examples of established biopsychosocial aging research exist. The biopsychosocial model of disease stresses the interconnectedness between biological, psychological and social factors in determining an individual’s medical condition. The interplay between the three factors determines the cause, manifestation and resolution of disease, emphasizing complex dynamics beyond biological factors. Aging research should therefore consider social and behavioral factors alongside biological factors to deal with the disruptive effects of the aging process. Complex pathways of psychosocial concepts on health are described and empirically tested, for instance, in the field of views on aging and health as well as relationship quality in health and aging.

### Socioeconomic inequalities and biological aging

Recent efforts for a better understanding of unequal aging have led to increased interest in the interplay between cumulative socioeconomic inequalities and biological aging [7, 11, 25]. Socioeconomic disadvantage throughout the life course poses a risk for the quality of aging and even accelerated biological aging, yet the mechanisms driving such differences have seldomly been explored in geroscience. Potential pathways between socioeconomic disadvantages and health outcomes could be mediated by biological processes, such as accelerated biological aging. The EU funded Lifepath Research Consortium aimed to investigate the underlying biological mechanisms through which socioeconomic inequalities would affect deterioration in healthy aging [25]. Drawing on omics data in combination with socioeconomic and behavioral information from 17 cohorts, the consortium looked at socioeconomically stratified biological markers, allostatic load and DNA methylation. The results of the studies suggest that socioeconomic circumstances are embedded in our biology from the outset and therefore affect aging processes across the life course.

It is reasonable to presume that the social context is closely linked to biological aging, particularly considering the intersection of multiple social inequalities, such as economic position, gender and ethnicity and accumulated over the life course. Methodological advances in social and behavioral gerontology include the multilevel analysis of individual heterogeneity and discriminatory accuracy (MAIHDA), which enables the investigation of intersectional effects of social inequalities on health outcomes. These advances could be valuable bridges to biogerontology, enabling the unravelling of how inequality gets under the skin through the aging process. Concerning MAIHDA, the study by Holman et al. [10] constitutes a scarce example where intersectional social inequalities in biomarkers of aging were investigated.

### Behavior and biological aging

It is widely recognized that behavioral factors, such as physical activity, healthy diet and stress management are modulators of the aging process and age-related health outcomes [20]. For instance, physical exercise was found to be associated with accelerated telomere shortening, indicating that behavior could accelerate cellular aging [29]; however, studies that investigate particular mechanisms of how behavior that translates into biological aging are adopted and maintained in the specific context are scarce. Such experience-dependent and context-dependent processes could be studied in light of how behavioral causation is mediated at the biological level. Ecologic momentary assessments (EMA) have largely been used in behavioral sciences. There have been some EMA studies using for example salivary biomarkers of stress response as well as physical activity assessed via accelerometry in combination with self-report measures (e.g., beliefs and affects that precede behavior), but their adoption in accelerated aging research is lacking [30]. The emergence of epigenetics is a valuable tool to understand how different behavioral factors interact at different life stages to modulate the rate of biological aging through altered epigenomes [2, 19, 20]. Although there is great potential, behavioral theory and principles have rarely been incorporated into geroscience [19].

### Social exchange processes and biological aging

It appears to be common sense that social embeddedness and health are connected, yet the specific pathways that drive this association from societal to individual levels can be very complex. The framework of Berkman et al. outlines the pathways from macro-level factors, which condition the extent, shape and nature of social networks at a mezzo-level, which, in turn provide opportunities for psychosocial mechanisms at the micro-level [4]. Psychosocial mechanisms include social support, social influence and engagement, but also person to person contacts and access to resources [4]. These psychosocial mechanisms were assumed to impact health through behavioral (e.g., diet, exercise), psychological (e.g., self-efficacy, sense of well-being) and physiological (e.g., allostatic load, immune system function) pathways.

How social exchange processes relate to biological aging was more explicitly investigated in studies from Kiecolt-Glaser’s group [13, 27]. They investigated married couples and how social support is associated with aging-related biomarkers (i.e., proinflammatory cytokines and insulin-like growth factor 1) [27]. More comprehensively, Kiecolt-Glaser et al. [13] described the potential pathways from chronic marital distress to mental health problems, such as depression, which then increase the risk for obesity, metabolic syndrome, and cardiovascular and cardiometabolic diseases through behavioral (e.g., poor diet, sleep problems, low exercise levels) to physiological (e.g., insulin, triglycerides), which also increases the likelihood of gut dysbiosis and accelerated aging (e.g., inflamm*aging*).

### Views on aging and biological aging

According to the stereotype embodiment theory (SET) proposed by Levy, positive and negative age stereotypes in our societies become embodied through internalization processes [16]. These stereotypes can be conscious or unconscious, but become salient from self-relevance (i.e., societal cues that prompt “old age”). Importantly, internalized age stereotypes are assumed to impact health through behavioral (e.g., sedentary behavior, medication adherence), psychological (e.g., expectations, which also alter behavioral responses such as cognitive and physical functioning) and physiological (e.g., increased cardiovascular and hypothalamic-pituitary-adrenal [HPA] responses to stress) pathways [16]. Wurm et al. differentiated age stereotypes, self-perceptions of aging, subjective age and personality and developmental processes that are assumed to lead to health-related outcomes via behavioral, psychological and physiological pathways (in line with the SET) and suggest differentiating between gain versus loss-related views on aging [28].

The evidence linking biomarkers of aging with views on aging was reviewed by Schönstein et al. [22]. They identified studies that associated views on aging with telomere length, blood-based biomarkers (e.g., C‑reactive protein [CRP], cystatin C), and functioning (grip strength, walking speed or cognitive performance). While this is an emerging field of research, the body of evidence is still scarce.

## Critical perspectives on geroscience from social sciences

Geroscience formulates a vision for preventing age-related diseases and loss of function by targeting biological mechanisms of aging [21]. There is a critical debate in social gerontology regarding the geroscience perspective on the aging process, but also on the need for incorporation of senescence into social science and social gerontology [8, 9]. Gilleard and Higgs, for instance, argued that recognizing the social structuring of later life is important to understand aging beyond “aging as senescence” including positive views on aging, benefits and gains for the aged person, the community and the society [9]. Social gerontology can critically capture concerns with anti-aging and longevity extension scenarios that have been raised by geroscience scholars. Conversely, ignoring senescence in social gerontology risks not comprehensively understanding aging in its interdependence of “ageing bodies, ageing subjects and ageing societies” [9].

## General conclusion

The aforementioned “vehicle” approach to use the findings of behavioral science as a method to ensure adherence and to initiate behavior in geroscience attempts, so to speak as a methodological tool, without these sciences themselves being part of the theory-testing or generating scientific process, must certainly be critically questioned. Research on an equal level between disciplinary perspectives would certainly be a much more fruitful approach. This approach would involve developing and testing both behavioral and geroscience theories at the same time or, in the best-case scenario, establishing even a truly interdisciplinary theory that integrates both perspectives into one approach.

This article briefly outlines existing research on behavior, inequality, social exchange, and views on aging in relation to the research on biology of aging; however, there are many more emerging fields of interaction that could not be touched on in the article. For instance, the lifespan theory by Carstensen et al. (e.g., [5]), but also established concepts such as loneliness [15], cognition, stress appraisal, and controls beliefs and their links to biogerontology are not discussed here for reasons of space limitations. Additionally, the rapidly expanding field of sociogenomics is likely to advance the inclusion of social factors into biological aging research by untangling complex interactions between inherent genetic susceptibility and social environmental exposures [31]. Finally, trends in population aging led to the emergence of biodemography, a promising field that revealed new insights into the nature of human longevity, including postponement of mortality and senescence, healthy life span and demography of healthy aging [32].

There are methodological advances in social and behavioral gerontology such as MAIHDA and EMA as mentioned before, which could incorporated into an interdisciplinary research agenda. Another important discussion refers to data infrastructures that enable this interdisciplinary work between behavioral and social gerontology and biogerontology. Concerning longitudinal and representative data, the health and retirement study is currently among the rare exceptions.

A joint research agenda that integrates all disciplinary approaches on an equal footing is necessary in order to successfully pursue this interdisciplinary endeavor of combining social and behavioral research with the biology of aging to answer the complex questions that concern our societies. The question remains, what can one perspective do better than the other and where synergies can best arise. This work provides examples of fruitful approaches and provides empirical examples but also shows the limitations.

The discussion should certainly be based on the negotiation of central terminologies as to what is understood as “normal aging” and what is understood as “pathological aging” or what can be understood as health and illness. The integration of social and behavioral science thinking with geroscience could also mean first agreeing on common questions that are of interest in aging: These could be the life span approach, conception of resilience and prevention, or understanding diversity, understanding plasticity and understanding the multidimensionality of any development or prediction of healthy life span or mortality. In this respect, the boundaries between social and behavioral science and geroscience seem to have to been overcome before cooperation with the same view on the aging process and a common view what constitutes a human is possible. In this respect, it has to be kept in mind that geroscience is not the same as biogerontology or biology of aging and objecting to one does not necessarily mean criticizing the other.

In this article a taxonomy of interdisciplinary models is proposed (see Fig. 1). This may structure existing theoretical and empirical work in gerontology and guide future attempts to work across disciplinary borders. Being clear about the nature or relation of the combination of constructs of models from different disciplines is the key to further joint research.

## Practical conclusion

While the recent attempts to move geroscience from basic science to clinical trials and broadening the disciplinary spectrum to successfully proceed with this translation, an equal and reciprocal interdisciplinary research agenda that incorporates social science and geroscience approaches would be needed.

## Declarations

For this article no studies with human participants or animals were performed by any of the authors. All studies mentioned were in accordance with the ethical standards indicated in each case.

## References

[1] Baltes PB (1997) On the incomplete architecture of human ontogeny: Selection, optimization, and compensation as foundation of developmental theory. Am Psychol 52:366 9109347 10.1037/0003-066X.52.4.366

[2] Belsky DW, Baccarelli AA (2023) To promote healthy aging, focus on the environment. Nat Aging: 1–11 10.1038/s43587-023-00518-7 PMC12459207 37946045

[3] Ben-Shlomo Y, Cooper R, Kuh D (2016) The last two decades of life course epidemiology, and its relevance for research on ageing. Int J Epidemiol 45:973–988 27880685 10.1093/ije/dyw096 PMC5841628

[4] Berkman LF, Glass T, Brissette I, Seeman TE (2000) From social integration to health: Durkheim in the new millenium. Soc Sci Med 51:843–857 10972429 10.1016/S0277-9536(00)00065-4

[5] Carstensen LL, Fung HH, Charles ST (2003) Socioemotional selectivity theory and the regulation of emotion in the second half of life. Motiv Emot 27:103–123 10.1023/A:1024569803230

[6] Crimmins EM (2020) Social hallmarks of aging: Suggestions for geroscience research. Ageing Reseach Rev 63:101136 10.1016/j.arr.2020.101136 PMC7530044 32798771

[7] Ferraro KF, Shippee TP (2009) Aging and cumulative inequality: how does inequality get under the skin? Gerontologist 49:333–343 19377044 10.1093/geront/gnp034 PMC2721665

[8] Fletcher JR (2020) Anti-aging technoscience & the biologization of cumulative inequality: Affinities in the biopolitics of successful aging. J Aging Stud 55:100899 33272453 10.1016/j.jaging.2020.100899 PMC7576313

[9] Gilleard C, Higgs P (2023) Ageing without senescence: A critical absence in social gerontology? J Aging Stud 66:101166 37704269 10.1016/j.jaging.2023.101166

[10] Holman D, Salway S, Bell A (2020) Mapping intersectional inequalities in biomarkers of healthy ageing and chronic disease in older English adults. Sci Rep 10:13522 32782305 10.1038/s41598-020-69934-8 PMC7419497

[11] Karimi M, Castagne R, Delpierre C et al (2019) Early-life inequalities and biological ageing: a multisystem Biological Health Score approach in UnderstandingSociety. J Epidemiol Community Health 73:693–702 30944170 10.1136/jech-2018-212010 PMC6678052

[12] Kennedy BK, Berger SL, Brunet A et al (2014) Geroscience: linking aging to chronic disease. Cell 159:709–713 25417146 10.1016/j.cell.2014.10.039 PMC4852871

[13] Kiecolt-Glaser JK, Wilson SJ, Madison A (2019) Marriage and Gut (Microbiome) Feelings: Tracing Novel Dyadic Pathways to Accelerated Aging. Psychosom Med 81:704–710 30308579 10.1097/PSY.0000000000000647 PMC6458105

[14] Kunkel SR, Settersten R Jr (2021) Aging, society, and the life course. Springer

[15] Lam JA, Murray ER, Yu KE et al (2021) Neurobiology of loneliness: a systematic review. Neuropsychopharmacology 46:1873–1887 34230607 10.1038/s41386-021-01058-7 PMC8258736

[16] Levy B (2009) Stereotype embodiment: A psychosocial approach to aging. Curr Dir Psychol Sci 18:332–336 20802838 10.1111/j.1467-8721.2009.01662.x PMC2927354

[17] Lopez-Otin C, Blasco MA, Partridge L et al (2013) The hallmarks of aging. Cell 153:1194–1217 23746838 10.1016/j.cell.2013.05.039 PMC3836174

[18] Lopez-Otin C, Blasco MA, Partridge L et al (2023) Hallmarks of aging: An expanding universe. Cell 186:243–278 36599349 10.1016/j.cell.2022.11.001

[19] Moffitt TE (2020) Behavioral and Social Research to Accelerate the Geroscience Translation Agenda. Ageing Res Rev 63:101146 32814128 10.1016/j.arr.2020.101146 PMC7894048

[20] Picard M (2011) Pathways to aging: the mitochondrion at the intersection of biological and psychosocial sciences. J Aging Res 2011:814096 21961065 10.4061/2011/814096 PMC3180824

[21] Rolland Y, Sierra F, Ferrucci L et al (2023) Challenges in developing Geroscience trials. Nat Commun 14:5038 37598227 10.1038/s41467-023-39786-7 PMC10439920

[22] Schönstein A, Trares K, Wahl H‑W (2022) Subjective Views of Aging and Objective Aging Biomarkers: Achievements and Questions in an Emerging Research. Area In: Subj Views Aging: Theory Res Pract Springer P: 153–168

[23] Sierra F (2016) The Emergence of Geroscience as an Interdisciplinary Approach to the Enhancement of Health Span and Life Span. Cold Spring Harb Perspect Med 6:a25163 26931460 10.1101/cshperspect.a025163 PMC4817738

[24] Skou ST, Mair FS, Fortin M et al (2022) Multimorbidity. Nat Rev Dis Primers 8:48 35835758 10.1038/s41572-022-00376-4 PMC7613517

[25] Vineis P, Avendano-Pabon M, Barros H et al (2017) The biology of inequalities in health: the LIFEPATH project. LLCS 8:118 10.14301/llcs.v8i4.448 PMC7235337 32478023

[26] Wahl H‑W, Heyl V (2015) Gerontologie-Einführung und Geschichte. Kohlhammer

[27] Wilson SJ, Bailey BE, Malarkey WB, Kiecolt-Glaser JK (2021) Linking Marital Support to Aging-Related Biomarkers: Both Age and Marital Quality Matter. J Gerontol B Psychol Sci Soc Sci 76:273–282 31428790 10.1093/geronb/gbz106 PMC7813187

[28] Wurm S, Diehl M, Kornadt AE et al (2017) How do views on aging affect health outcomes in adulthood and late life? Explanations for an established connection. Dev Rev 46:27–43 33927468 10.1016/j.dr.2017.08.002 PMC8081396

[29] Rebelo-Marques A, De Sousa Lages A, Andrade R (2018) Aging Hallmarks: The Benefits of Physical Exercise. Front Endocrinol 20(9):258 10.3389/fendo.2018.00258 PMC5980968 29887832

[30] Pauly T, Michalowski VI, Nater UM, Gerstorf D, Ashe MC, Madden KM, Hoppmann CA (2019) Everyday associations between older adults’ physical activity, negative affect, and cortisol. Health Psycholgy 38(6):494–501 10.1037/hea0000743 31008643

[31] Joo YY, Cha J, Freese J, Hayes MG (2022) Cognitive Capacity Genome-Wide Polygenic Scores Identify Individuals with Slower Cognitive Decline in Aging. Genes 13(8):1320 35893057 10.3390/genes13081320 PMC9331374

[32] Vaupel J (2010) Biodemography of human ageing. Nature 464:536–542 20336136 10.1038/nature08984 PMC4010874

